# Metacognition as a tool to differentiate cognitively unimpaired from impaired subjects: judgments of learning in prospective memory task

**DOI:** 10.1002/alz.092334

**Published:** 2025-01-03

**Authors:** Augusto J. Mendes, Federica Ribaldi, Christian Chicherio, Mathilde Bastien, Stefano F Cappa, Matthias Kliegel, Giovanni B. Frisoni

**Affiliations:** ^1^ Laboratory of Neuroimaging of Aging (LANVIE), University of Geneva, Geneva Switzerland; ^2^ Laboratory of Neuroimaging of Aging, University of Geneva, Geneve Switzerland; ^3^ Geneva University Hospitals, Geneva Switzerland; ^4^ Université de Genève, Psychologie et Scieces de l'éducation, Geneve Switzerland; ^5^ IRCCS San Giovanni di Dio Fatebenefratelli, Brescia Italy; ^6^ Laboratory of Cognitive Aging, University of Geneva, Geneva Switzerland; ^7^ Laboratory of Neuroimaging of Aging, University of Geneva, Geneva Switzerland

## Abstract

**Background:**

Metacognition may give useful insight in early stages of Alzheimer’s disease (AD) continuum. Recent studies suggest differences in the ability of judging their own cognitive performance along clinical stages. For instance, subjects with mild cognitive impairment (MCI) overestimate, whereas people with subjective cognitive decline (SCD) tend to underestimate their cognitive performance. One cognitive function compromised in prodromal AD is prospective memory (PM), which refers to the ability to remember something that has to be done. However, the metacognitive abilities in PM tasks are still not clear when comparing individuals with varying degrees of cognitive impairment.

**Method:**

Our study included 94 subjects from the Geneva Memory Center which are cognitively unimpaired (CU; n = 53), SCD (n = 26), and MCI (n = 15). All subjects performed a PM task with judgements of learning (JOL) to evaluate their metacognition. For that, participants were instructed to memorize a list of pairs of words and to press a specific key if the first word of the pair appeared in a posterior lexical decision task. A one‐way ANOVA tested the differences in PM performance and corresponding JOLs among the three clinical groups. Lastly, the association of PM metacognition with subjective complaints and anxiety levels was tested using Pearson correlations.

**Result:**

The PM ability was significantly different among clinical groups (F = 10.5, p<0.001). Post‐hoc analysis revealed that MCI showed significantly lower PM in comparison with CU (p<0.001) and SCD (p = 0.003). Moreover, the JOL of PM also differed among the three groups (F = 4.5, p = 0.02), namely the MCI group showed lower JOLs in comparison with CU (p = 0.04). Likewise, correlations suggested a significant association between PM‐JOLs and subjective complaints (self‐other; R = 0.33, p = 0.006), whereas the association with anxiety was not statistically significant (p>0.05).

**Conclusion:**

Our study suggested differences in the PM and its JOL between MCI and CU/SCD. However, our study was not able to differentiate between the CU and SCD groups at the PM and the corresponding JOL, even though their difference was significantly correlated with subjective functioning.

